# Exploring Gender Diversity in Transgender and Non-Binary Adults Accessing a Specialized Service in Italy

**DOI:** 10.3390/healthcare11152150

**Published:** 2023-07-28

**Authors:** Marta Mirabella, Bianca Di Giannantonio, Guido Giovanardi, Irene Piras, Alessandra D. Fisher, Vittorio Lingiardi, Luca Chianura, Jiska Ristori, Anna Maria Speranza, Alexandro Fortunato

**Affiliations:** 1 Department of Dynamic and Clinical Psychology, and Health Studies, Sapienza University of Rome, 00185 Rome, Italy; bianca.digiannantonio@uniroma1.it (B.D.G.); guido.giovanardi@uniroma1.it (G.G.); piras.1535319@studenti.uniroma1.it (I.P.); vittorio.lingiardi@uniroma1.it (V.L.); annamaria.speranza@uniroma1.it (A.M.S.); alexandro.fortunato@uniroma1.it (A.F.); 2 Andrology, Women’s Endocrinology and Gender Incongruence Unit, Florence University Hospital, 50100 Florence, Italy; alessandra.fisher@gmail.com (A.D.F.); jiskaristori@gmail.com (J.R.); 3 Gender Identity Development Service, Hospital S. Camillo-Forlanini, 00152 Rome, Italy; chianuraluca@gmail.com

**Keywords:** gender identity, access to care, gender expression, gender non-conforming adults

## Abstract

In Italy, studies investigating gender identity and expression in gender non-conforming adults are lacking, as well as data regarding the non-binary population. The present study aimed at dimensionally exploring how transgender and non-binary Italian adults identify and express their gender. The Gender Diversity Questionnaire (GDQ) was administered to a sample of 109 adult subjects aged 18-60 years accessing a gender-specialized service in Rome. The majority of the participants were aged 18–24 years (52.3%), whereas fewer subjects were aged 25–35 years (33%) and 35 years and older (14.7%). Most participants (84.4%) identified themselves as trans binary, while the remaining (15.6%) identified as non-binary. Trans binary participants reported a stable gender identity, whereas non-binary participants reported a more fluid gender identity over time and across contexts. Younger subjects recognized the use of chosen names, pronouns, and clothes as important for their gender expression, whereas older subjects attributed more importance to physical appearance and emotions. Differences regarding gender-affirmative interventions emerged between non-binary and trans binary participants. The findings evidence that gender non-conforming adults accessing gender-specialized services have unique needs and features, and thus it is essential to shed light on this population by providing greater visibility and recognition.

## 1. Introduction

Over the past decade, the field of gender studies has increased in visibility, and the dimensionality and diversity of gender identity have achieved greater recognition. Moreover, the greater visibility of a wide variety of gender non-conforming identities has led to a redefinition of gender. Accordingly, gender is now considered a spectrum, rather than a binary confined to only male and female categories [1]. Moreover, it is no longer viewed as innate, but as an identity construction. This changing context has also facilitated the emergence of individuals expressing and experiencing gender in a range of ways across the spectrum [2]. Specifically, several studies have shown an increase in the number of individuals who do not conform to the traditional gender binary (i.e., male vs. female) and instead identify with a non-binary identity [3,4,5]. The term non-binary is used to indicate individuals who do not exclusively identify as either male or female, but instead identify along the gender spectrum. Some non-binary persons move between binary genders (i.e., genderfluid*),* while others do not identify with any gender (i.e., agender, no gender, genderless), and still others identify simultaneously and/or alternately with two (i.e., bigender), three (i.e., trigender), or more (i.e*.,* polygender) gender identities or feel only a partial connection to one gender identity (i.e., demigender). The heterogeneity of non-binary identities evidences a variability of trajectories and developmental paths in this population compared to transgender binary subjects (i.e., people who perceive their gender identity in a binary way, identifying as transgender men and women). Indeed, non-binary individuals acquire awareness of their identity usually not until late adolescence or early adulthood, compared to transgender binary subjects where identity awareness usually occurs around the early stages of puberty [6,7,8]. Despite the increase of non-binary identities, only a small percentage of non-binary individuals seek assistance from specialized gender services. Vincent’s study of non-binary subjects found that they tended to avoid expressing their gender identity in some settings (e.g., specialized services), due to fear of stigma and discrimination [9]. A lack of sociocultural visibility, added to a dearth of awareness and information within systems promoting a gender dichotomy, may represent a further barrier to the elaboration and affirmation of non-binary identities [7,10].

In addition to the increase of different gender identities, internationally, several studies have described a rise in the population accessing specialized gender services over the past few years [11,12,13,14,15].

In Italy, the framework concerning specialized gender services is diversified and complex. All the centers belong to the National Health Service and are linked to the Osservatorio Nazionale sull’Identità di Genere (National Observatory on Gender Identity), which, together with other scientific societies, networks various professionals involved in supporting the health of trans people. These societies promote specific guidelines that centers usually adopt, consisting of a psychodiagnostic assessment to evaluate gender diversity, general psychological functioning and medical affirming treatments which comprise puberty suppression for eligible adolescents (which so far has only been applied to a small number of adolescents under 16 years of age), cross-sex hormone therapy for adolescents 16 years of age and older, and gender reassignment surgery (GRS) for adults (18 years of age and older), if requested.

The distribution of specialized services throughout the country is diversified. Most centers are in urban areas (e.g., Rome, Naples), despite being situated in different regions of the country often far apart, and there is a lack of centers on islands (e.g., Sicily and Sardinia). In some regions, there are also several LGBTQIA+ associations and nonprofit organizations which provide social and psychological support, especially to families, but do not provide hormone therapy. The great distances between regions and the expensive connections between the mainland and the islands make it very difficult for many people to access the centers.

Regarding the Italian population of specialized gender services, some research has noted a broad heterogeneity of the transition paths and a considerable increase in the number of youths identifying as gender diverse and seeking assessment, support, and treatment, whereas there is a lack of evidence regarding gender non-conforming adults (i.e., older than 18 years old) accessing specialized gender services [16,17,18,19].

Notably, gender non-conforming adults seeking gender care may perceive specific gender identity-related concerns regarding authenticity, self-understanding, coming out, and medical transition processes [20,21]. In addition, based on the age of access to specialized centers, individuals who seek gender-affirming treatments in adulthood may have perceived, prior to access, different experiences regarding their gender and sexual identity. These experiences may comprise the decision-making process regarding their coming out, questioning with regard to their gender identity, puberty and bodily-related experiences, fantasies and desires about medicalized and affirming interventions, and expectations regarding the future [22,23]. Previous studies [21,24,25,26,27,28,29,30,31,32,33,34] on adult transgender populations in Italy have investigated several factors, including psychological and psychopathological correlates and experiences with hormones and sexuality, but only a few have focused on gender identity among gender non-conforming adults. None, to our knowledge, have investigated gender identity characteristics (e.g., gender fluidity; gender categories) and gender identity-related features (e.g., social transition; gender expression; medical affirming interventions) in adult samples accessing specialized gender services.

Against this backdrop, the present study is proposed as a continuum of previous research conducted by Mirabella et al. [17], which examined gender identity and expression among adolescents accessing an Italian specialized gender service. Building upon this foundation, the present study has been conducted within the same gender-specialized service, but with a focus on the adult population seeking care, which has received limited attention in the existing literature.

Specifically, the aim of the present study was to explore the gender non-conforming adult population accessing a specialized gender service in Italy, investigating gender categories, gender expression, and age-related differences in the sample, shedding light on a population that is still poorly understood. In line with the literature [9], we hypothesized a limited number of non-binary individuals compared to trans binary subjects accessing the service and also assumed that there will be a difference in the age distribution of adults accessing the specialized service. Specifically, we expected to observe a higher proportion of younger adults seeking care compared to older adults. Building on previous research [14], we also hypothesized that there will be differences among the adult population based on age with regard to social transition, gender expression, and gender identity. Specifically, we assumed that younger subjects will perceive a more fluid identity and place greater importance on identity-related aspects (e.g., clothes, names, and pronouns) in relation to their gender expression compared to adult subjects. Additionally, we hypothesized that there will be a higher percentage of young adults who have undergone social transition compared to older adults.

## 2. Material and Methods

The present study utilized the Gender Diversity Questionnaire (GDQ) [2]. The GDQ aims at exploring gender identity, gender expression, and factors influencing gender expression as multidimensional, multifactorial, and changing constructs across time and life contexts. Gender identity is investigated through open-ended (i.e., how would you describe your gender identity today) and categorical questions, which ask respondents to describe their gender identity and identify the gender categories to which they feel they belong. Additionally, multidimensional questions explore gender fluidity, specifically the extent to which respondents experience their gender identity as fixed and unchanging over time. Greater fluidity consists in respondents’ affirmation to be exploring their gender identity and experiencing context- or time-based gender identity changes. Regarding gender expression, open-ended (i.e., which aspects are most important to you in the way you express your gender) and categorical questions explore the factors which respondents consider most important and influential in their gender expression. Finally, categorical questions investigate the factors that influence respondents’ experiences regarding their gender.

### 2.1. Participants

The sample included all individuals between the ages of 18 and 60 who accessed the Gender Identity Development Service (SAIFIP) in Rome between May 2020 and December 2021. A total of 109 gender non-conforming adults were considered. The inclusion criterion for this study was to be aged 18 years or older.

### 2.2. Procedure

The study was submitted for approval to the Ethical Committee of the Department of Dynamic and Clinical Psychology, and Health Studies of Sapienza University of Rome (Protocol number: N. 327, 16 April 2020). An informed consent form that explained the aim of the research was filled in by all the participants.

### 2.3. Data Analysis

The distribution of variables in the sample was assessed through descriptive and frequency analyses. Differences between age groups and between trans binary and non-binary gender categories regarding gender identity and gender fluidity were assessed using chi-square tests. Subsequently, Braun and Clarke’s thematic analysis [35] was implemented to recognize themes in the open-ended questions, reflecting key aspects of participants’ experiences. Two of the authors identified points of interest and created a set of codes. Subsequently, the authors developed a list of themes and sub-themes. Further on, two different authors read and cross-checked the codes to confirm that the themes were correctly developed. Finally, the themes and sub-themes were named.

## 3. Results

### 3.1. Demographics

The sample consisted of adult individuals aged 18 to 60 years old. The mean age of the sample was 26.5 years (*SD* = 8.65). To evaluate age-related differences among subjects, we divided participants into three age groups (Table 1) following Arnett’s study [36] evidencing that there are specific differences in age stages during adulthood. Specifically, individuals aged between 18 and 24 face several challenges such as exploring their identity, having to self-focus, and experiencing feelings of transition, as well as dealing with the possibilities of transformation. Conversely, older individuals aged 25–35 encounter other life challenges such as establishing long-term relationships, having to achieve personal and financial stability, balancing multiple roles, and having to adjust to the responsibilities and expectations associated with adulthood. Moreover, individuals older than 35 years old often have to deal with prioritizing their physical and mental well-being, engaging in activities that promote health, managing stress, and adapting to changes associated with aging, and often focus on stabilizing and consolidating their careers, seeking opportunities for growth. Thus, we aimed to investigate how individuals in these different age groups position themselves with respect to gender identity and expression.

Specifically, 52.3% (*n* = 57) of the sample were aged 18–24 years, 33.03% (*n* = 36) were aged 25–35 years, and 14.7% (*n* = 16) were aged 35 years and older. Regarding educational level, 18.3% (*n* = 20) were educated through secondary school, 50.5% (*n* = 55) were educated through high school, 11.0% (*n* = 12) had attended (or were attending) a professional school, and 18.3% (*n* = 20) had attended (or were attending) university. One subject reported attending another type of school and one subject did not report their educational level. Thirteen subjects (11.9%) reported having dropped out of school. Among the experiences and reasons for failure, participants described bullying, difficulty studying, and personal issues.

With respect to employment, 34.9% (*n* = 38) of the subjects were students, 31.2% (*n* = 34) were employed, and 29.4% (*n* = 32) were unemployed. Five subjects did not specify their employment. Concerning household composition, 56% (*n* = 61) of the subjects lived with family, 15.6% (*n* = 17) lived alone, 9.2% (*n* = 10) lived with a partner or cohabitant, 3.7% (*n* = 4) lived with a partner and children, 1.8% (*n* = 2) lived with friends, 11% (*n* = 12) reported living in a context other than those mentioned above, and 3% (*n* = 3) did not specify their household composition.

Most subjects (94.5%; *n* = 103) reported that they had not begun hormone therapy, while six subjects (5.50%) reported undertaking hormones. Regarding psychotherapy, 60.6% (*n* = 66) reported having started psychotherapy prior to accessing the specialized gender service, while 50.5% (*n* = 55) reported having started psychotherapy after accessing the service.

### 3.2. Gender Identity

Gender identity was evaluated by asking subjects to indicate one or more gender categories that best described them. The proposed categories comprised several labels, such as male, female, trans non-binary, agender, and undefined identifications, as well as no identification, reflecting a conception of gender as a spectrum. Specifically, 84.4% of the sample (*n* = 92) used the words “trans” and “male”, “trans” and “female”, or only “male”/“female” to describe their gender identity. These subjects were included in the trans binary identity category. Moreover, 15.6% (*n* = 17) defined themselves as non-binary, 2.8% (*n* = 3) defined themselves as agender, 5.54% (*n* = 6) did not identify with any category, and 2.8% (*n* = 3) described themselves as undefined. Respondents who defined themselves as non-binary, agender, any category, and undefined were included in the non-binary category. Across the present study, participants identifying with the trans binary and the non-binary categories will be compared to evaluate differences with regard to gender expression and gender identity. However, distinctions between these categories have been described to display the different gender categories with which participants identify themselves.

**Figure 1:**
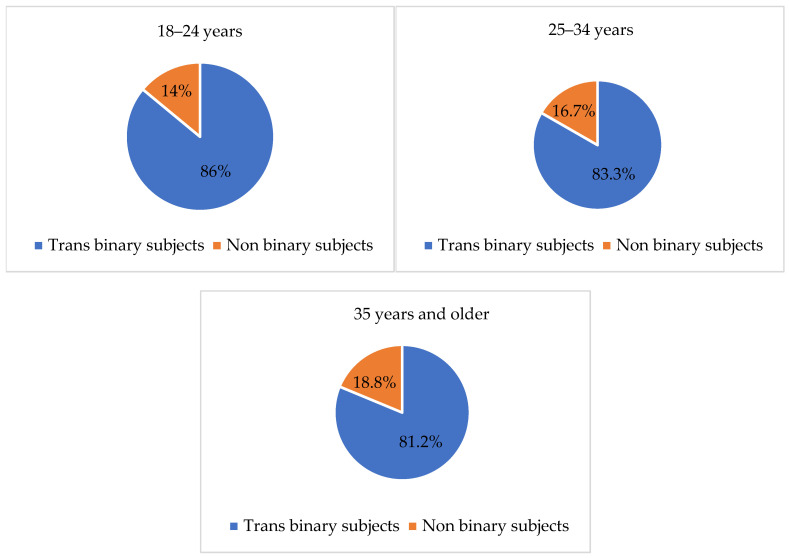
Trans binary and non-binary subjects by age group.

With regard to the distribution of trans binary and non-binary subjects among the three age groups (i.e., 18–24 years, 25–34 years, 35 years and older), no significant differences emerged from the chi-square test. However, as Figure 1 shows, most participants in all age groups defined themselves as trans binary.

### 3.3. Gender Fluidity

Gender fluidity was investigated using the items of the GDQ [2] between the three age groups and between the trans binary and non-binary subjects to evaluate to what extent individuals experienced gender fluidity. No statistically significant differences emerged with regard to the age group comparison. Nevertheless, Table 2 evidences statistically significant differences in three out of the four dimensions of gender fluidity when comparing the trans binary and non-binary categories. As expected, non-binary subjects appeared to be more fluid over time and in different contexts. Moreover, significantly more non-binary participants reported that they were currently investigating their gender identity.

Specifically, for the item “*My gender identity is fluid, and it changes in different contexts*”, 92.4% of trans binary participants reported “never”, 5.4% reported “sometimes”, and 1.1% reported “always” and one subject did not answer; meanwhile in the non-binary group, 58.8% reported “never”, 35.3% reported “sometimes”, and 5.9% reported “always”. Additionally, in response to the item “*My gender identity is fluid, it changes over time*”, 91.3% of trans binary people reported “never”, 7.6% reported “sometimes”, and none stated “always” and one subject did not answer; in comparison, 17.6% of non-binary participants stated “always”, 35.3% reported “sometimes”, and 47.1% reported “never”. Finally, with regard to the item “*I am currently exploring my gender identity*”, 6.5% of trans binary subjects reported “always”, compared to 41.2% of non-binary subjects, and 66.3% of trans binary responded “never”, compared to 23.5% of non-binary subjects.

### 3.4. Questioning Gender Identity

**Figure 2:**
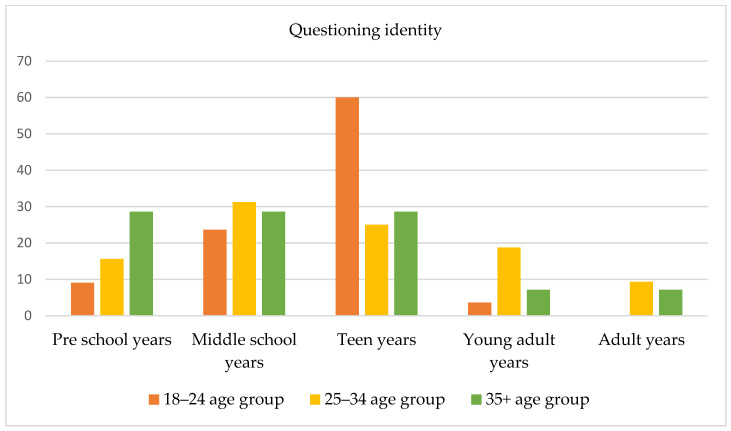
Age at which subjects began to question their gender identity.

The age at which subjects began to question their gender identity before accessing the gender-specialized service was also investigated; 101 responses were obtained and were divided into five age periods: (1) preschool years (aged 3–5 years), (2) middle school years (aged 6–11 years), (3) teen years (aged 12–18 years), (4) young adult years (aged 18–25 years), and (5) adult years (aged 25 years and older). No significant differences among groups emerged from the chi-square test. As Figure 2 shows, younger subjects (i.e., 18–24 years old) typically reported that they had started to question their gender identity during their teen years; only a low percentage (9.09%) reported that such questioning had begun during their preschool years. Most subjects aged 24–35 years reported that they had started to question their gender identity in middle school (31.25%) and during their young adult years (18.75%). Of note, compared to younger subjects, a higher percentage of participants in this age group reported questioning their gender identity during their preschool years (15.65%). Finally, a small percentage of subjects aged 35 years and older reported that they had started to question their gender identity during their young adult years, and a high percentage (28.57%) reported that they had begun this questioning during school (i.e., middle school and teen years) and preschool years (28.57%).

Subjects were also asked open-ended questions regarding how they identified prior to questioning their gender identity. Of the subjects who responded, 4.30% reported that they had identified as “misunderstood”, 10.75% reported that they “did not think about it”, and 13.97% reported “not knowing how to identify themselves”. Also, one subject reported, “I identified as a boy, but my parents tried to convert me”. Of note, some subjects reported that they had identified as lesbian or gay prior to questioning their gender identity. Specifically, 6.45% of subjects had identified as lesbian and 3.22% of subjects had identified as gay.

### 3.5. Social Transition

**Figure 3:**
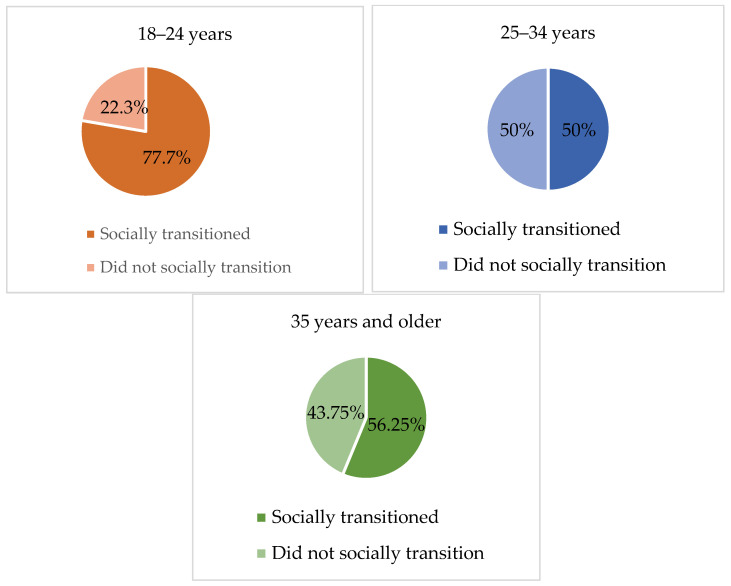
Social transition by age group.

Considering the fact that the sample comprised adult individuals, we questioned if they had socially transitioned prior to accessing specialized gender services and, if so, at what age. Social transition refers to the adoption of one’s preferred name, pronoun, gender expression (e.g., clothes, haircut), and/or gender roles in correspondence to one’s perceived gender identity. Overall, 104 responses were collected and of these subjects, 65.38% (*n =* 68) reported having socially transitioned, whereas 34.62% (*n =* 36) reported that they had not socially transitioned. With regard to social transition among age groups, no statistically significant differences emerged from the chi-square test. As Figure 3 evidences, 77.7% (*n* = 42) of subjects aged 18–24 years socially transitioned prior to accessing the specialized gender service, whereas 50% (*n* = 17) of participants aged 25–34 years, and 56.25% (*n* = 9) of those aged 35 years and older, confirmed that they had socially transitioned prior to being referred to the specialized gender service. Such results provide evidence that early adults were more likely to have transitioned before accessing the gender-specialized service compared to older adults.

### 3.6. Influential Factors

**Figure 4:**
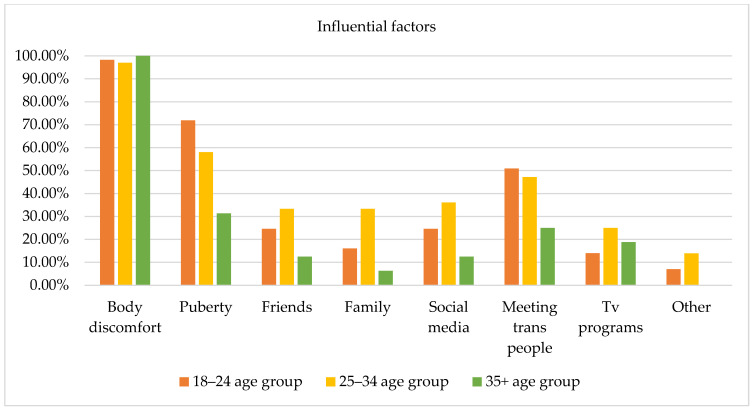
Factors influencing gender identity by age group.

Several factors of the GDQ (i.e., body uneasiness, puberty, friends, family, social media, TV programs, and connections with trans individuals) that could potentially influence how participants experienced their gender were evaluated via a yes/no checklist and open-ended questions and 108 responses were collected (i.e., one subject did not express any preferences) [2]. Figure 4 displays the influential factors among the three age groups; however, no statistically significant differences emerged from the chi-square test.

Body discomfort was the factor that most influenced how participants experienced their gender (i.e., 98.2% of subjects aged 18–24 years, 97.2% of subjects aged 25–34 years old, and 100% of subjects aged 35 years and older), evidencing that body distress represents a specific type of influence for individuals accessing a specialized service, triggering reflections and questions regarding one’s gender identity. The second most influential factor was pubertal development, especially among subjects aged 18–24 years (71.9%), compared to subjects aged 35 years and older (31.3%). This result may evidence that for young adults, experiences associated with puberty are not so far back in time, and puberty may have involved significant body and social dysphoria influencing how participants experienced their gender. Connections with other trans people appeared as a fundamental factor for both subjects aged 18–24 years (50.9%) and 25–34 years (47.2%). Furthermore, use of social media (e.g., YouTube, Instagram, Facebook) was more relevant for subjects aged 25–34 years (36.1%) than for subjects aged 18–24 years (24.6%) and 35 years and older (12.5%). Finally, support from friends and family was identified as a factor of influence, especially by subjects aged 18–24 years and subjects aged 25–34 years compared to subjects aged 35 years and older.

In the open-ended questions, subjects aged 18–24 years indicated the following aspects as influential for their gender expression: internet forums (1.8%); not identifying with their biological sex (1.8%); and literature, music, cinematography, and video games (1.8%). Subjects aged 25–34 years indicated literature, music, cinematography, and video games (5.7%); self-reflection (2.8%); not being recognized according to their gender identity (2.8%); and discovering the drag world (2.8%). Finally, subjects aged 35 years and older did not report any other factors that influenced their gender expression.

**Figure 5:**
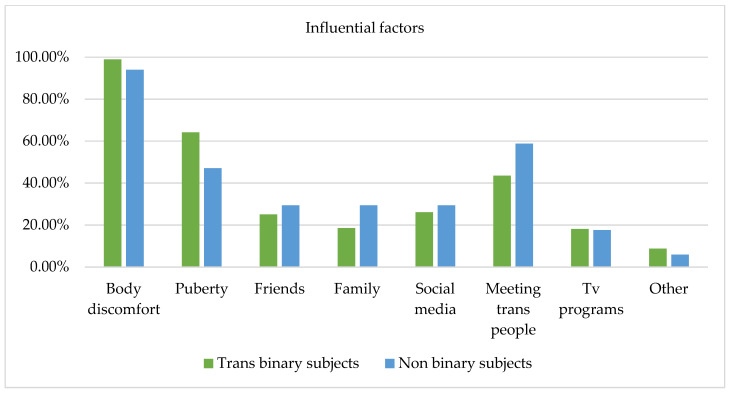
Factors influencing gender identity in trans binary and non-binary subjects.

Figure 5 evidences influential factors among trans binary and non-binary individuals. The chi-square test revealed no statistically significant differences between these groups. Body discomfort emerged as the most influential factor for 100% of the non-binary participants and 98.9% of the trans binary participants. Thus, body distress seems to be a key aspect in both groups, evidencing how body perception specifically influences gender identity across gender categories. Moreover, a higher percentage of trans binary subjects (64.1%) compared to non-binary subjects (47.10%) indicated pubertal development as a factor of influence in the experience of gender. Connections with trans people appeared to be more important for non-binary subjects (58.8%) than for trans binary subjects (43.5%). Use of social media and support from friends and family were reported as influential for both groups, but especially for non-binary subjects (29.4%). TV programs also had the same level of influence for both groups. It seems evident that in the process of understanding and constructing one’s gender identity, these tools can facilitate introspection, especially for non-binary individuals who have to struggle with invisibility and can share their experiences through social networks.

Regarding other factors of influence, trans binary participants indicated the following: internet forums (1.1%); literature, music, cinematography, and video games (2.17%); not identifying with their biological sex (1.1%); not being recognized according to their gender identity (1.1%); self-reflection (1.1%); discovering the drag world (1.1%); and unspecified factors (1.1%). Non-binary participants indicated only literature, music, cinematography, and video games (5.88%).

### 3.7. Gender Expression

**Figure 6:**
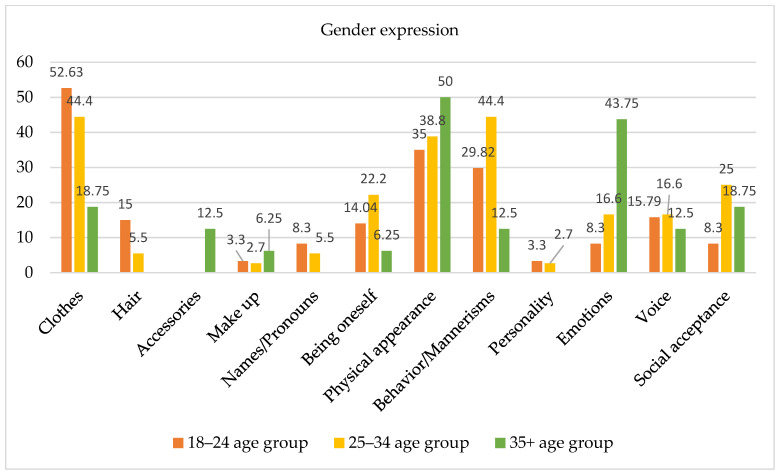
Factors contributing to gender expression by age group.

Open-ended questions were also used to ask participants to describe which aspects they evaluated as essential for their gender expression. Considering the large age span of the sample, we found it interesting to evaluate which features are considered important among the three age groups (see Figure 6), even though no statistically significant differences emerged between the groups from the chi-square analysis. Subjects aged 18–24 years described clothing as a key factor for their gender expression (52.63%). Moreover, these respondents underlined the role of physical appearance (35%), particularly body shape, as well as body-related behaviors such as breast binding, hiding the male physique, and growing hair. In this regard, one subject noted, “Since I’ve been wearing padding in my boxers, I’m more peaceful, knowing that something is visible in the crotch of my pants”; another claimed, “I put socks in my underwear”. Also, this age group identified behavior and mannerisms (29.82%) (i.e., attitude) as important, as well as the use of chosen names and pronouns (8.3%).

Among subjects aged 25–35 years, behavior and mannerisms (44.4%) and clothing (44.4%) appeared to be important factors influencing gender expression. Additionally, physical appearance (38.8%) and body-related behaviors (particularly breast binding) were identified as key factors. Moreover, 16.6% of subjects in this age group identified emotions as being important.

Finally, subjects aged 35 years and older identified body and physical appearance (50%) as important influential factors for gender expression. Also, emotions (43.75%) such as affection and empathy were identified as more important for gender expression than they were for the other two age groups. Conversely, the use of chosen names and pronouns was not named as an important factor by this age group.

### 3.8. Medical Interventions

**Figure 7:**
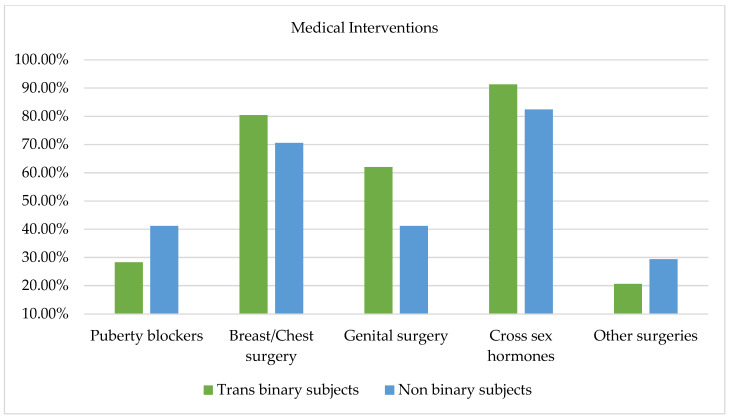
Desired medical interventions in trans binary and non-binary subjects.

Gender-affirmative interventions were investigated among participants asking them which intervention they wished to undergo from a checklist including puberty blockers, breast/chest surgery, genital surgery, and cross-sex hormone therapy. Overall, 108 responses were collected. As Figure 7 evidences, from the comparison between trans binary and non-binary participants, no significant differences emerged between the two groups; however, it is worth highlighting that a greater proportion of non-binary subjects (41.2%) reported a desire to receive puberty blockers relative to trans binary participants (28.3%). Similar percentages of trans binary (91.3%) and non-binary (82.4%) subjects expressed a desire to undergo cross-sex hormone therapy. Similarly, a consistent number of trans binary (80.4%) and non-binary (70.6%) subjects expressed a desire to undergo breast and chest surgeries. Conversely, more trans binary subjects (62%) than non-binary subjects (41.2%) expressed a desire to undergo genital surgery. Finally, as Figure 7 evidences, some participants also filled in an open-ended question with other desired surgeries not comprised in the checklist. Among the responses, 8.69% of trans binary subjects expressed a desire to undergo an ovariectomy and hysterectomy, and 10.86% expressed a desire to undergo other kinds of surgeries such as facial femininization, Adam’s apple reduction, rhinoplasty, and vocal cord intervention. In contrast, only a few non-binary participants expressed a desire to pursue other interventions; specifically, two subjects expressed a desire to undergo a hysterectomy and two desired facial femininization.

**Figure 8:**
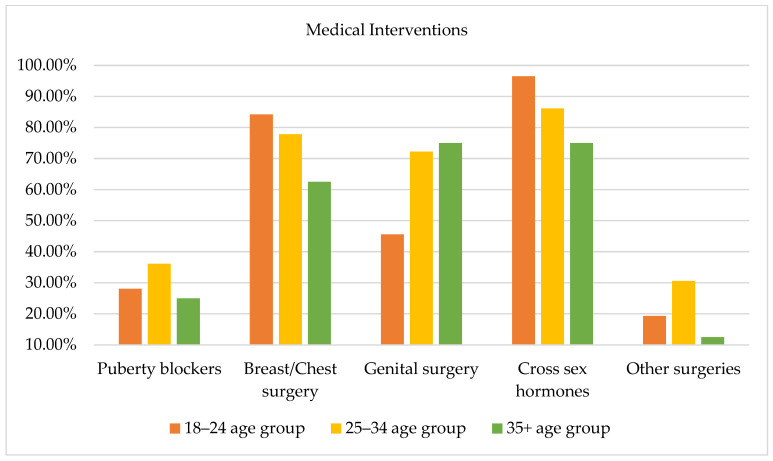
Desired medical interventions by age group.

Figure 8 illustrates the comparison among the three age groups with regard to desired medical interventions and no statistically significant differences emerged. With regard to the use of puberty blockers among the three age groups, it is worth underlining that the desire to undertake such therapy was higher among subjects aged 25–34 years (36.10%) compared to subjects aged 18–24 (28.10%) years and subjects aged 35 years and older (25%). Regarding the use of cross-sex hormone therapy, subjects aged 18–25 years (96.5%) compared to both groups of older subjects expressed a desire to undertake this type of therapy. Regarding physical interventions, a high percentage in each group expressed willingness to pursue breast and chest surgeries. Moreover, a considerable percentage of subjects aged 25–34 (72.20%) and subjects aged 35 years and older (75%) expressed a desire to undergo genital surgeries. It is noteworthy that some participants expressed the desire to undergo other desired interventions not included in the checklist. Specifically, as Figure 8 evidences, subjects aged 24–35 years highlighted willingness to undergo surgeries related to non-secondary sexual characteristics (e.g., facial femininization, rhinoplasty, vocal cord interventions). On the other hand, subjects aged 18–24 years expressed a desire to undergo specific gender-affirming interventions, including the removal of the uterus or ovaries.

## 4. Discussion

To our knowledge, this is the first study that has explored a sample of gender non-conforming adults accessing a specialized gender service in Italy, with respect to their experiences and perceptions of gender. As hypothesized, most of the sample was comprised of trans binary individuals. Such a result is consistent with previous research addressing gender non-conforming youths [17], evidencing that overall, trans binary individuals are more likely to seek gender-affirming interventions compared to non-binary subjects. This finding may be related to the fact that non-binary individuals may fear facing specific challenges in healthcare contexts, including feeling misunderstood and invalidated by specialists with a binary view of gender, and may perceive health professionals as not prepared to address their unique needs [5,37,38,39]. In addition, and in line with research on the adolescent sample [17], significantly more non-binary adults described that they were currently investigating their gender identity and embracing a gender fluid identity. Thus, it seems evident that compared to trans binary individuals, non-binary individuals may consider constricting having to define themselves within specific gender categories, perhaps preferring to embrace a more fluid identity across time and contexts [6].

About the age of the sample, as hypothesized, most of the subjects were early adults. This result is in line with the literature [11,12,13,14,16,18,40,41] regarding specialized gender services, and highlights a declining trend in the age of individuals with gender diversity accessing specialized services. In this vein, results evidenced that early adults had typically started questioning their gender identity during their teen years. This finding is in line with Arnett’s study [36], which underlines that between the ages of 18 and 24, individuals tend to explore their identities, dealing with self-definition and individualization. This finding also suggests that, currently, younger individuals show a higher level of awareness regarding the nuances of identity and demonstrate a willingness to question whether their identity is fixed, concrete, and permanent, leaning towards a more evolving and fluid perspective. As a result, they may be more likely to refer to a specialized service. Moreover, this may suggest that younger generations are growing up in more accepting and affirming environments that encourage gender expression and identity exploration. Conversely, older subjects reported that they began questioning their gender identity during their preschool years. This finding highlights a time gap between the period when older subjects started questioning their gender identity and the time at which they actually approached a specialized gender service. Considering the later age of referral, it can be assumed that older participants grew up in contexts that did not encourage their gender expression and thus they may have encountered resistances identifying with specific labels they desired for themselves, experiencing difficulties in revealing their sexual and gender identity.

These results also suggest a change in the onset of gender incongruence, with late-onset gender incongruence (i.e., during or after puberty) becoming more common than previously documented in the literature which describes predominantly early-onset gender incongruence (i.e., before puberty) [42]. Interestingly, regarding questioning of gender identity, our results evidenced that some subjects reported that they had identified as lesbian or gay before identifying within trans binary and non-binary categories. We can assume that for some gender non-conforming adults, coming out as lesbian or gay may have been a way to embrace and deal with feelings of being gender diverse and/or a first step towards later coming out as trans binary or non-binary.

Also, results showed that most subjects had socially transitioned prior to accessing the specialized gender services. This finding may indicate that although participants accessed a specialized service during adulthood, they managed to socially transition, accomplishing an initial move towards self-actualization and self-recognition of their gender identity as a first footstep prior to gender-affirming interventions. These findings are aligned with previous research [43,44,45] showing that individuals who undergo social transition, who are open about their trans status, and who live in an area where their identified gender is recognized, are more likely to seek gender-affirming care and support from specialized services. Moreover, as hypothesized, most of the individuals who socially transitioned were early adults, which may suggest that younger generations often experience more accepting and inclusive environments than older generations. Indeed, although Italy still faces challenges in achieving full inclusiveness for transgender people, who experience social, legal, and health discrimination, societal attitudes toward gender diversity are evolving, especially in younger generations, who may therefore feel more comfortable and supported in openly expressing their gender identity [46,47,48]. In addition, we can hypothesize that young adults make greater use of the Internet, thus having access to resources and information about gender identity (e.g., through social media), and that this accessibility can facilitate the recognition of their identity, as the Internet can be a space for support, awareness, sharing, and affirmation.

Furthermore, results evidenced that the way subjects perceived and experienced their gender non-conformity was influenced by many factors, including the distress around body, puberty development, support from friends and family, and the role of social media and relations with the LGBTQI+ community. Specifically, body discomfort was identified as a key factor of influence for all the participants. This result is aligned with findings regarding adolescent individuals, showing that also adult subjects which refer to specialized gender services perceive significant body discomfort which may include dissatisfaction with specific body parts, discomfort with body size, and dissatisfaction with one’s appearance [17]. In this regard, pubertal development was described as a crucial factor influencing participants’ experience of gender, especially among early adults and trans binary subjects. This finding is consistent with the results of previous studies [17,49,50,51] highlighting that, for trans binary individuals, the experience of puberty seems associated with high levels of discomfort, since it may remind them of their gender incongruence. Specifically, the development of marked gender characteristics occurring with puberty can generate higher rates of distress and body dissatisfaction among individuals who desire to transition toward a binary gender, relative to non-binary subjects. The fact that puberty did not emerge as a significantly influential factor for older adults may be explained by their later age, perhaps because they had more time to signify and come to terms with their gender identity and pubertal development, relative to younger subjects. Also, in line with findings on the adolescent population, support from friends and family was indicated as an influential factor for gender identity, especially among early adults [17]. This result is also aligned with previous research showing that family support can be a protective factor for transgender individuals [52].

Specifically, early adults may have defined family support as a crucial factor of influence because, at the time of referral, they may be still living with the family, as in Italy, individuals tend to leave their family home at a later age relative to individuals in other countries [53].

In addition, in line with the literature [54], participants described the connections to trans people as a key factor of influence, underlining the importance of the sense of belonging to a community.

As evidenced, of the variety of features influencing gender expression, our participants reported physical appearance and specific related behaviors (e.g., hiding certain body parts) as key factors, suggesting that they may represent the primary way in which gender non-conforming adults define and express themselves. Thus, we can assume that the role of appearance, especially for individuals seeking treatment, may be crucial with regard to gender expression, representing the primary trigger of body and social dysphoria. Moreover, the use of chosen names and preferred pronouns was identified as an important factor in their gender expression, especially by early adults. This may indicate that among younger adults, the use of inclusive language comprising chosen names and pronouns is significant in the process of seeking recognition and affirmation of their gender identity [55]. Conversely, compared to early adults, older subjects were more likely to identify emotions such as sensibility and happiness as important for their gender expression, underlining the importance of feeling good about themselves. This finding may be related to the fact that, as portrayed by Arnett [36], during adulthood, especially from 25 years old, individuals tend to develop greater emotional maturity and self-awareness which may lead them to focus on emotional well-being as they strive for personal growth and contentment.

This result is also in line with previous studies [56] evidencing that gender non-conforming individuals may perceive significant distress with regards to their gender identity and such discomfort can impact on their day-to-day lives. Thus, feeling positive emotions may be an important influential factor with regard to the way they manage their gender expression.

Moreover, regarding medical interventions, young adults expressed more desire to undergo gender-affirming interventions compared to older adults. This result may be related to the fact that older adults may be aware that undergoing medical interventions at a later age has several implications and lower chances of a good outcome from an aesthetic point of view.

Also, trans binary participants underlined a desire to undergo treatments regarding the removal of primary and secondary sexual characteristics, confirming the narrative that the experience of trans binary individuals is focused on a deep desire to obtain the primary sex characteristics of another gender than one’s assigned sex [57]. Conversely, non-binary subjects showed greater interest in interventions pertaining to secondary sexual characteristics. Notably, non-binary individuals may not express a specific gender transition trajectory, wishing to navigate their own identities without approaching a specialized gender service, reporting different needs with respect to hormone and gender-affirmative therapies compared to trans binary subjects [58].

As gender is no longer considered an unquestionable concept, but increasingly considered a changeable identity construction, it is essential to shed light on and expand our understanding of different aspects and issues experienced by individuals accessing specialized gender services. Indeed, the process of navigating one’s gender identity may differ between individuals in different stages of life and it can also depend on their cultural context, which might affect not only the development of these services but also the way people perceive their gender identity and eventually lead to psychological stress. Hence, there is a need to explore gender incongruence not only diagnostically, but also through the lens of subjective experience, in order to better understand the factors that may contribute to psychological distress, the social determinants of gender incongruence, and the ways in which gender is expressed and perceived, in order to tailor treatments to the specific needs of each individual.

## 5. Limitations

The present study has some limitations that need to be acknowledged. First, because the sample was recruited from only one specialized gender service, the results may not provide a complete picture of the variety of identifications among the population of adults accessing specialized gender services in Italy. Also, the number of individuals identifying as non-binary may have been underrepresented, due to the relative lack of non-binary subjects in clinical settings. Moreover, as all participants were at the beginning of their assessment, it was predictable that gender-related issues influencing participants’ perception and expression of gender identity were significantly present. Finally, a limitation of this study concerns the possible susceptibility of the descriptive results, which cannot be generalized to the entire transgender and non-binary population.

## 6. Conclusions

As gender and identity become increasingly subjective and subjectivized, the need grows for a more thorough understanding of how gender non-conforming individuals seeking gender-affirming treatments experience their gender identity. Of note, gender non-conforming adults experience specific pathways with regard to their gender identity and expression and have different needs compared to gender non-conforming adolescents. Therefore, this study, by providing information regarding gender non-conforming adults accessing a specialized service in Italy, allows us to gain a broader perspective and a more profound knowledge of how these subjects express and perceive their gender identity. However, it is important to highlight that the present study is purely descriptive, as the findings offer valuable insights into the characteristics, experiences, and behaviors of the participants without, however, providing specific statistical differences. Nonetheless, the present study can contribute to expanding our understanding of the unique experiences and challenges faced by adults undergoing a gender transition, by exploring their narratives and perspectives and shedding light on the nuanced aspects of adult transition journeys that have not been extensively explored. Also, the present findings contribute to improving clinicians’ work by helping them understand the experiences and features of gender non-conforming adults, promoting interventions and care that prioritize the unique psychological well-being needs of adults during their gender transition paths.

## 7. Future Directions

Future research should aim to study larger samples of adults accessing specialized gender services to further examine age trends, gender identity, and gender expression. In addition, comparing samples of adult individuals accessing multiple specialized gender services would provide valuable insights into the broader adult population seeking support and care. Given the complex interplay of factors influencing the experience of gender non-conforming adult individuals accessing specialized gender services, future studies should also thoroughly explore body image concerns, the role of body dissatisfaction, and experiences with respect to gender-affirming surgeries in order to provide more targeted and effective interventions. In addition, further research should investigate the influence of gender roles, social and cultural norms, and how these aspects affect gender non-conforming adults and their experience of dysphoria. Finally, to gain a more comprehensive understanding of gender transition in adulthood, conducting longitudinal studies that follow participants over time would greatly benefit research.

## Figures and Tables

**Table 1:** Demographic characteristics.

**Age Group**	
18–24 years	52.3% (*n* = 57)
25–34 years	33% (*n* = 36)
35+ years	14.7% (*n* = 16)
**Marital status**	
Single	57.8% (*n* = 63)
In a relationship	33.9% (*n* = 36)
Married	5.5% (*n* = 6)
Cohabitating	1.8% (*n* = 2)
Separated/divorced	0.9 (*n* = 1)
**Educational level**	
Secondary school	18.3% (*n* = 20)
High school	50.5% (*n* = 55)
Professional school	11% (*n* = 12)
University	18.3% (*n* = 20)
Other	0.9 (*n* = 1)
**School drop-out**	
Not a school drop-out	85.3% (*n* = 93)
School drop-out	11.9% (*n* = 13)
**Employment**	
Student	34.9% (*n* = 38)
Employed	31.2% (*n* = 34)
Unemployed	29.4% (*n* = 32)
Not specified	4.59% (*n* = 5)
**Household composition**	
Living with family	56.0% (*n =* 61)
Living with friends	1.8% (*n* = 2)
Living with partner/cohabitant	9.2% (*n* = 10)
Living alone	15.6% (*n* = 17)
Living with partner and children	3.7% (*n* = 4)
Other	11% (*n* = 12)
**Family events**	
Parental divorce	34.9% (*n* = 38)
No parental divorce	60.6% (*n* = 66)
**Children**	
Yes	2.8% (*n* = 3)
No	97.2% (*n* = 106)
**Hormone therapy**	
Undergoing hormone therapy	5.54% (*n* = 6)
Not undergoing hormone therapy	94.5% (*n* = 103)
**Psychotherapy**	
Psychotherapy prior to arrival	60.6% (*n* = 66)
Psychotherapy after arrival	50.5% (*n* = 55)

**Table 2:** Results of a chi-square test comparing trans binary and non-binary participants on gender fluidity.

	Fixed Gender Identity (No Time- or Context-Based Change)		Fluid Gender Identity (Context-Based Change)		Fluid Gender Identity (Time-Based Change)		Currently Exploring Gender Identity	
	Chi2	*p*	Chi2	*p*	Chi2	*p*	Chi2	*p*
Trans binary vs. non-binary	10.516	0.005	16.206	<0.001 *	28.568	<0.001 *	19.125	<0.001 *

* *p* < 0.001.

## Data Availability

The data presented in this study are available on request from the corresponding author.
