# Correction: Mirabella et al. Exploring Gender Diversity in Transgender and Non-Binary Adults Accessing a Specialized Service in Italy. *Healthcare* 2023, *11*, 2150

**DOI:** 10.3390/healthcare13131593

**Published:** 2025-07-03

**Authors:** Marta Mirabella, Bianca Di Giannantonio, Guido Giovanardi, Irene Piras, Alessandra D. Fisher, Vittorio Lingiardi, Luca Chianura, Jiska Ristori, Anna Maria Speranza, Alexandro Fortunato

**Affiliations:** 1 Department of Dynamic and Clinical Psychology, and Health Studies, Sapienza University of Rome, 00185 Rome, Italy; 2 Andrology, Women’s Endocrinology and Gender Incongruence Unit, Florence University Hospital, 50100 Florence, Italy; 3 Gender Identity Development Service, Hospital S. Camillo-Forlanini, 00152 Rome, Italy

The sample size and data collection period of the original publication [1] were revised.

## Text Correction

Following a re-evaluation of the dataset and the application of updated inclusion criteria, the final sample size was adjusted from 112 to 109 participants. Accordingly, the abstract was revised to reflect this change:

**Abstract:** In Italy, studies investigating gender identity and expression in gender non-conforming adults are lacking, as well as data regarding the non-binary population. The present study aimed at dimensionally exploring how transgender and non-binary Italian adults identify and express their gender. The Gender Diversity Questionnaire (GDQ) was administered to a sample of 109 adult subjects aged 18-60 years accessing a gender-specialized service in Rome. The majority of the participants were aged 18–24 years (52.3%), whereas fewer subjects were aged 25–35 years (33%) and 35 years and older (14.7%). Most participants (84.4%) identified themselves as trans binary, while the remaining (15.6%) identified as non-binary. Trans binary participants reported a stable gender identity, whereas non-binary participants reported a more fluid gender identity over time and across contexts. Younger subjects recognized the use of chosen names, pronouns, and clothes as important for their gender expression, whereas older subjects attributed more importance to physical appearance and emotions. Differences regarding gender-affirmative interventions emerged between non-binary and trans binary participants. The findings evidence that gender non-conforming adults accessing gender-specialized services have unique needs and features, and thus it is essential to shed light on this population by providing greater visibility and recognition.

Additionally, the data collection period was corrected to accurately reflect the study timeline: initially reported as “between January 2019 and December 2021”, it has been revised to “between May 2020 and December 2021”. A correction has been made to “Section 2. Material and Methods, 2.1. Participants”, concerning both the number of participants and the data collection period.

*2.1. Participants*

The sample included all individuals between the ages of 18 and 60 who accessed the Gender Identity Development Service (SAIFIP) in Rome between May 2020 and December 2021. A total of 109 gender non-conforming adults were considered. The inclusion criterion for this study was to be aged 18 years or older.

Corrections have been made to “Section 3. Results, 3.1. Demographics, 3.2. Gender Identity, 3.3. Gender Fluidity, 3.4. Questioning Gender Identity, 3.5. Social Transition, 3.6. Influential Factors, 3.7. Gender Expression, 3.8. Medical Interventions” to reflect these revisions to the sample. As a result of the reduced sample size, percentage values were adjusted accordingly:

**3.** **Results**
*3.1.* *Demographics*

The sample consisted of adult individuals aged 18 to 60 years old. The mean age of the sample was 26.5 years (*SD* = 8.65). To evaluate age-related differences among subjects, we divided participants into three age groups (Table 1) following Arnett’s study [36] evidencing that there are specific differences in age stages during adulthood. Specifically, individuals aged between 18 and 24 face several challenges such as exploring their identity, having to self-focus, and experiencing feelings of transition, as well as dealing with the possibilities of transformation. Conversely, older individuals aged 25–35 encounter other life challenges such as establishing long-term relationships, having to achieve personal and financial stability, balancing multiple roles, and having to adjust to the responsibilities and expectations associated with adulthood. Moreover, individuals older than 35 years old often have to deal with prioritizing their physical and mental well-being, engaging in activities that promote health, managing stress, and adapting to changes associated with aging, and often focus on stabilizing and consolidating their careers, seeking opportunities for growth. Thus, we aimed to investigate how individuals in these different age groups position themselves with respect to gender identity and expression.

Specifically, 52.3% (*n* = 57) of the sample were aged 18–24 years, 33.03% (*n* = 36) were aged 25–35 years, and 14.7% (*n* = 16) were aged 35 years and older. Regarding educational level, 18.3% (*n* = 20) were educated through secondary school, 50.5% (*n* = 55) were educated through high school, 11.0% (*n* = 12) had attended (or were attending) a professional school, and 18.3% (*n* = 20) had attended (or were attending) university. One subject reported attending another type of school and one subject did not report their educational level. Thirteen subjects (11.9%) reported having dropped out of school. Among the experiences and reasons for failure, participants described bullying, difficulty studying, and personal issues.

With respect to employment, 34.9% (*n* = 38) of the subjects were students, 31.2% (*n* = 34) were employed, and 29.4% (*n* = 32) were unemployed. Five subjects did not specify their employment. Concerning household composition, 56% (*n* = 61) of the subjects lived with family, 15.6% (*n* = 17) lived alone, 9.2% (*n* = 10) lived with a partner or cohabitant, 3.7% (*n* = 4) lived with a partner and children, 1.8% (*n* = 2) lived with friends, 11% (*n* = 12) reported living in a context other than those mentioned above, and 3% (*n* = 3) did not specify their household composition.

Most subjects (94.5%; *n* = 103) reported that they had not begun hormone therapy, while six subjects (5.50%) reported undertaking hormones. Regarding psychotherapy, 60.6% (*n* = 66) reported having started psychotherapy prior to accessing the specialized gender service, while 50.5% (*n* = 55) reported having started psychotherapy after accessing the service.

*3.2.* *Gender Identity*

Gender identity was evaluated by asking subjects to indicate one or more gender categories that best described them. The proposed categories comprised several labels, such as male, female, trans non-binary, agender, and undefined identifications, as well as no identification, reflecting a conception of gender as a spectrum. Specifically, 84.4% of the sample (*n* = 92) used the words “trans” and “male”, “trans” and “female”, or only “male”/“female” to describe their gender identity. These subjects were included in the trans binary identity category. Moreover, 15.6% (*n* = 17) defined themselves as non-binary, 2.8% (*n* = 3) defined themselves as agender, 5.54% (*n* = 6) did not identify with any category, and 2.8% (*n* = 3) described themselves as undefined. Respondents who defined themselves as non-binary, agender, any category, and undefined were included in the non-binary category. Across the present study, participants identifying with the trans binary and the non-binary categories will be compared to evaluate differences with regard to gender expression and gender identity. However, distinctions between these categories have been described to display the different gender categories with which participants identify themselves.

With regard to the distribution of trans binary and non-binary subjects among the three age groups (i.e., 18–24 years, 25–34 years, 35 years and older), no significant differences emerged from the chi-square test. However, as Figure 1 shows, most participants in all age groups defined themselves as trans binary.

*3.3.* *Gender Fluidity*

Gender fluidity was investigated using the items of the GDQ [2] between the three age groups and between the trans binary and non-binary subjects to evaluate to what extent individuals experienced gender fluidity. No statistically significant differences emerged with regard to the age group comparison. Nevertheless, Table 2 evidences statistically significant differences in three out of the four dimensions of gender fluidity when comparing the trans binary and non-binary categories. As expected, non-binary subjects appeared to be more fluid over time and in different contexts. Moreover, significantly more non-binary participants reported that they were currently investigating their gender identity.

Specifically, for the item “*My gender identity is fluid, and it changes in different contexts*”, 92.4% of trans binary participants reported “never”, 5.4% reported “sometimes”, and 1.1% reported “always” and one subject did not answer; meanwhile in the non-binary group, 58.8% reported “never”, 35.3% reported “sometimes”, and 5.9% reported “always”. Additionally, in response to the item “*My gender identity is fluid, it changes over time*”, 91.3% of trans binary people reported “never”, 7.6% reported “sometimes”, and none stated “always” and one subject did not answer; in comparison, 17.6% of non-binary participants stated “always”, 35.3% reported “sometimes”, and 47.1% reported “never”. Finally, with regard to the item “*I am currently exploring my gender identity*”, 6.5% of trans binary subjects reported “always”, compared to 41.2% of non-binary subjects, and 66.3% of trans binary responded “never”, compared to 23.5% of non-binary subjects.

*3.4.* *Questioning Gender Identity*

The age at which subjects began to question their gender identity before accessing the gender-specialized service was also investigated; 101 responses were obtained and were divided into five age periods: (1) preschool years (aged 3–5 years), (2) middle school years (aged 6–11 years), (3) teen years (aged 12–18 years), (4) young adult years (aged 18–25 years), and (5) adult years (aged 25 years and older). No significant differences among groups emerged from the chi-square test. As Figure 2 shows, younger subjects (i.e., 18–24 years old) typically reported that they had started to question their gender identity during their teen years; only a low percentage (9.09%) reported that such questioning had begun during their preschool years. Most subjects aged 24–35 years reported that they had started to question their gender identity in middle school (31.25%) and during their young adult years (18.75%). Of note, compared to younger subjects, a higher percentage of participants in this age group reported questioning their gender identity during their preschool years (15.65%). Finally, a small percentage of subjects aged 35 years and older reported that they had started to question their gender identity during their young adult years, and a high percentage (28.57%) reported that they had begun this questioning during school (i.e., middle school and teen years) and preschool years (28.57%).

Subjects were also asked open-ended questions regarding how they identified prior to questioning their gender identity. Of the subjects who responded, 4.30% reported that they had identified as “misunderstood”, 10.75% reported that they “did not think about it”, and 13.97% reported “not knowing how to identify themselves”. Also, one subject reported, “I identified as a boy, but my parents tried to convert me”. Of note, some subjects reported that they had identified as lesbian or gay prior to questioning their gender identity. Specifically, 6.45% of subjects had identified as lesbian and 3.22% of subjects had identified as gay.

*3.5.* *Social Transition*

Considering the fact that the sample comprised adult individuals, we questioned if they had socially transitioned prior to accessing specialized gender services and, if so, at what age. Social transition refers to the adoption of one’s preferred name, pronoun, gender expression (e.g., clothes, haircut), and/or gender roles in correspondence to one’s perceived gender identity. Overall, 104 responses were collected and of these subjects, 65.38% (*n =* 68) reported having socially transitioned, whereas 34.62% (*n =* 36) reported that they had not socially transitioned. With regard to social transition among age groups, no statistically significant differences emerged from the chi-square test. As Figure 3 evidences, 77.7% (*n* = 42) of subjects aged 18–24 years socially transitioned prior to accessing the specialized gender service, whereas 50% (*n* = 17) of participants aged 25–34 years, and 56.25% (*n* = 9) of those aged 35 years and older, confirmed that they had socially transitioned prior to being referred to the specialized gender service. Such results provide evidence that early adults were more likely to have transitioned before accessing the gender-specialized service compared to older adults.

*3.6.* *Influential Factors*

Several factors of the GDQ (i.e., body uneasiness, puberty, friends, family, social media, TV programs, and connections with trans individuals) that could potentially influence how participants experienced their gender were evaluated via a yes/no checklist and open-ended questions and 108 responses were collected (i.e., one subject did not express any preferences) [2]. Figure 4 displays the influential factors among the three age groups; however, no statistically significant differences emerged from the chi-square test.

Body discomfort was the factor that most influenced how participants experienced their gender (i.e., 98.2% of subjects aged 18–24 years, 97.2% of subjects aged 25–34 years old, and 100% of subjects aged 35 years and older), evidencing that body distress represents a specific type of influence for individuals accessing a specialized service, triggering reflections and questions regarding one’s gender identity. The second most influential factor was pubertal development, especially among subjects aged 18–24 years (71.9%), compared to subjects aged 35 years and older (31.3%). This result may evidence that for young adults, experiences associated with puberty are not so far back in time, and puberty may have involved significant body and social dysphoria influencing how participants experienced their gender. Connections with other trans people appeared as a fundamental factor for both subjects aged 18–24 years (50.9%) and 25–34 years (47.2%). Furthermore, use of social media (e.g., YouTube, Instagram, Facebook) was more relevant for subjects aged 25–34 years (36.1%) than for subjects aged 18–24 years (24.6%) and 35 years and older (12.5%). Finally, support from friends and family was identified as a factor of influence, especially by subjects aged 18–24 years and subjects aged 25–34 years compared to subjects aged 35 years and older.

In the open-ended questions, subjects aged 18–24 years indicated the following aspects as influential for their gender expression: internet forums (1.8%); not identifying with their biological sex (1.8%); and literature, music, cinematography, and video games (1.8%). Subjects aged 25–34 years indicated literature, music, cinematography, and video games (5.7%); self-reflection (2.8%); not being recognized according to their gender identity (2.8%); and discovering the drag world (2.8%). Finally, subjects aged 35 years and older did not report any other factors that influenced their gender expression.

Figure 5 evidences influential factors among trans binary and non-binary individuals. The chi-square test revealed no statistically significant differences between these groups. Body discomfort emerged as the most influential factor for 100% of the non-binary participants and 98.9% of the trans binary participants. Thus, body distress seems to be a key aspect in both groups, evidencing how body perception specifically influences gender identity across gender categories. Moreover, a higher percentage of trans binary subjects (64.1%) compared to non-binary subjects (47.10%) indicated pubertal development as a factor of influence in the experience of gender. Connections with trans people appeared to be more important for non-binary subjects (58.8%) than for trans binary subjects (43.5%). Use of social media and support from friends and family were reported as influential for both groups, but especially for non-binary subjects (29.4%). TV programs also had the same level of influence for both groups. It seems evident that in the process of understanding and constructing one’s gender identity, these tools can facilitate introspection, especially for non-binary individuals who have to struggle with invisibility and can share their experiences through social networks.

Regarding other factors of influence, trans binary participants indicated the following: internet forums (1.1%); literature, music, cinematography, and video games (2.17%); not identifying with their biological sex (1.1%); not being recognized according to their gender identity (1.1%); self-reflection (1.1%); discovering the drag world (1.1%); and unspecified factors (1.1%). Non-binary participants indicated only literature, music, cinematography, and video games (5.88%).

*3.7.* *Gender Expression*

Open-ended questions were also used to ask participants to describe which aspects they evaluated as essential for their gender expression. Considering the large age span of the sample, we found it interesting to evaluate which features are considered important among the three age groups (see Figure 6), even though no statistically significant differences emerged between the groups from the chi-square analysis. Subjects aged 18–24 years described clothing as a key factor for their gender expression (52.63%). Moreover, these respondents underlined the role of physical appearance (35%), particularly body shape, as well as body-related behaviors such as breast binding, hiding the male physique, and growing hair. In this regard, one subject noted, “Since I’ve been wearing padding in my boxers, I’m more peaceful, knowing that something is visible in the crotch of my pants”; another claimed, “I put socks in my underwear”. Also, this age group identified behavior and mannerisms (29.82%) (i.e., attitude) as important, as well as the use of chosen names and pronouns (8.3%).

Among subjects aged 25–35 years, behavior and mannerisms (44.4%) and clothing (44.4%) appeared to be important factors influencing gender expression. Additionally, physical appearance (38.8%) and body-related behaviors (particularly breast binding) were identified as key factors. Moreover, 16.6% of subjects in this age group identified emotions as being important.

Finally, subjects aged 35 years and older identified body and physical appearance (50%) as important influential factors for gender expression. Also, emotions (43.75%) such as affection and empathy were identified as more important for gender expression than they were for the other two age groups. Conversely, the use of chosen names and pronouns was not named as an important factor by this age group.

*3.8.* *Medical Interventions*

Gender-affirmative interventions were investigated among participants asking them which intervention they wished to undergo from a checklist including puberty blockers, breast/chest surgery, genital surgery, and cross-sex hormone therapy. Overall, 108 responses were collected. As Figure 7 evidences, from the comparison between trans binary and non-binary participants, no significant differences emerged between the two groups; however, it is worth highlighting that a greater proportion of non-binary subjects (41.2%) reported a desire to receive puberty blockers relative to trans binary participants (28.3%). Similar percentages of trans binary (91.3%) and non-binary (82.4%) subjects expressed a desire to undergo cross-sex hormone therapy. Similarly, a consistent number of trans binary (80.4%) and non-binary (70.6%) subjects expressed a desire to undergo breast and chest surgeries. Conversely, more trans binary subjects (62%) than non-binary subjects (41.2%) expressed a desire to undergo genital surgery. Finally, as Figure 7 evidences, some participants also filled in an open-ended question with other desired surgeries not comprised in the checklist. Among the responses, 8.69% of trans binary subjects expressed a desire to undergo an ovariectomy and hysterectomy, and 10.86% expressed a desire to undergo other kinds of surgeries such as facial femininization, Adam’s apple reduction, rhinoplasty, and vocal cord intervention. In contrast, only a few non-binary participants expressed a desire to pursue other interventions; specifically, two subjects expressed a desire to undergo a hysterectomy and two desired facial femininization.

Figure 8 illustrates the comparison among the three age groups with regard to desired medical interventions and no statistically significant differences emerged. With regard to the use of puberty blockers among the three age groups, it is worth underlining that the desire to undertake such therapy was higher among subjects aged 25–34 years (36.10%) compared to subjects aged 18–24 (28.10%) years and subjects aged 35 years and older (25%). Regarding the use of cross-sex hormone therapy, subjects aged 18–25 years (96.5%) compared to both groups of older subjects expressed a desire to undertake this type of therapy. Regarding physical interventions, a high percentage in each group expressed willingness to pursue breast and chest surgeries. Moreover, a considerable percentage of subjects aged 25–34 (72.20%) and subjects aged 35 years and older (75%) expressed a desire to undergo genital surgeries. It is noteworthy that some participants expressed the desire to undergo other desired interventions not included in the checklist. Specifically, as Figure 8 evidences, subjects aged 24–35 years highlighted willingness to undergo surgeries related to non-secondary sexual characteristics (e.g., facial femininization, rhinoplasty, vocal cord interventions). On the other hand, subjects aged 18–24 years expressed a desire to undergo specific gender-affirming interventions, including the removal of the uterus or ovaries.

In the seventh paragraph of “Section 4. Discussion”, the term “transpeople” was replaced with “trans people”, as the previous version contained a typographical error.

## Error in Figures/Tables

Several frequencies and percentages were updated following the revision of the dataset, resulting in the final sample size being adjusted from 112 to 109 participants. As a result, Table 1 and Table 2, as well as Figure 1, Figure 2, Figure 3, Figure 4, Figure 5, Figure 6, Figure 7 and Figure 8, have been updated to reflect the corrected data. The revised tables and figures appear below. 

The authors state that the scientific conclusions are unaffected. This correction was approved by the Academic Editor. The original publication has also been updated.

## Figures and Tables

**Figure 1:**
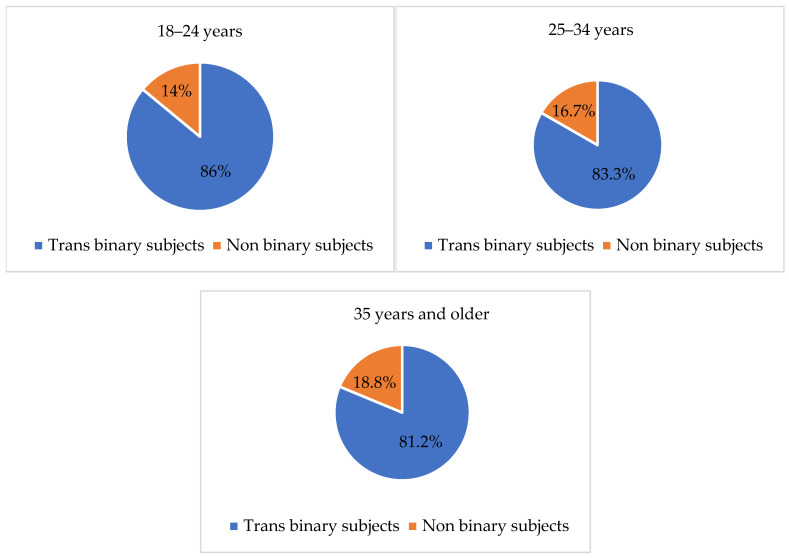
Trans binary and non-binary subjects by age group.

**Figure 2:**
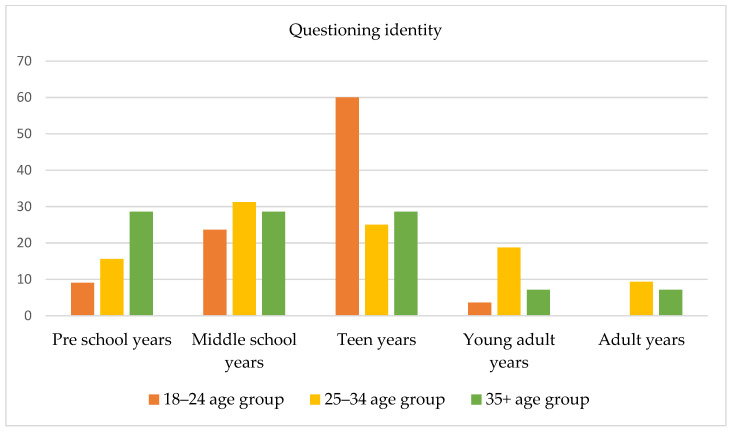
Age at which subjects began to question their gender identity.

**Figure 3:**
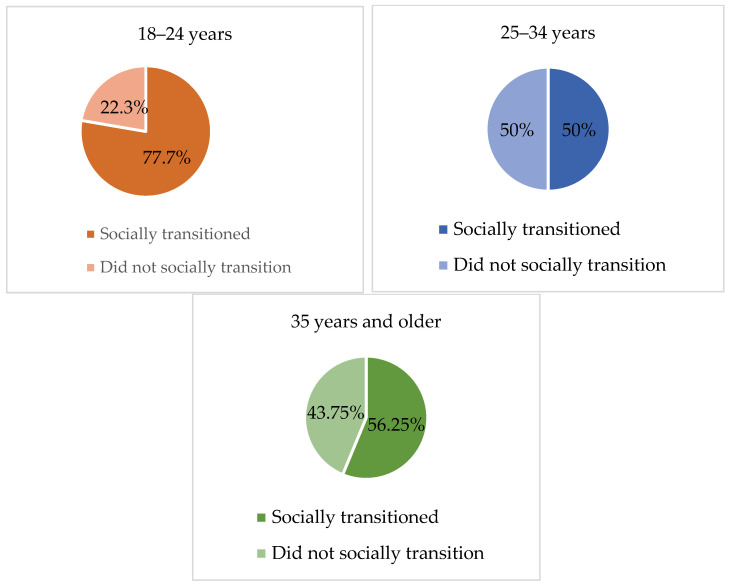
Social transition by age group.

**Figure 4:**
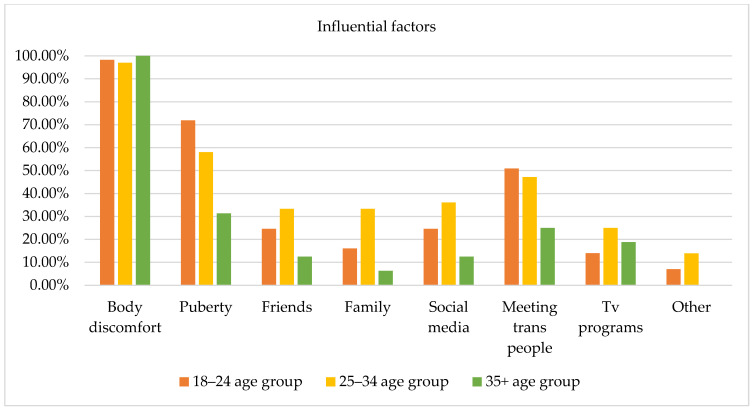
Factors influencing gender identity by age group.

**Figure 5:**
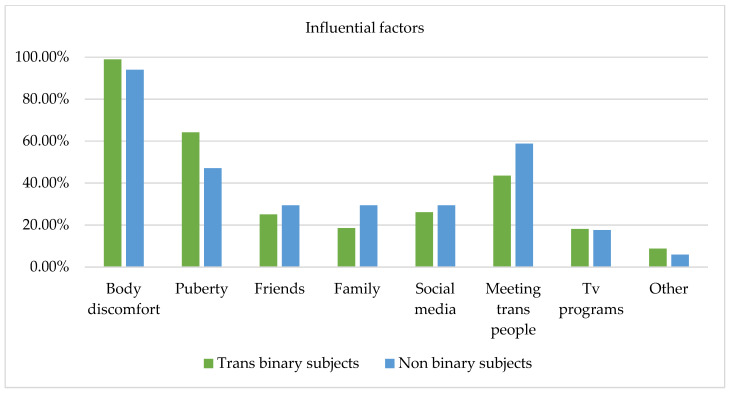
Factors influencing gender identity in trans binary and non-binary subjects.

**Figure 6:**
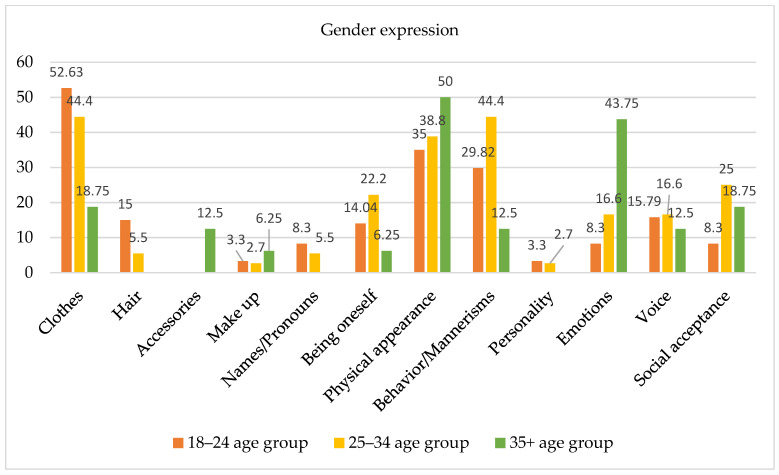
Factors contributing to gender expression by age group.

**Figure 7:**
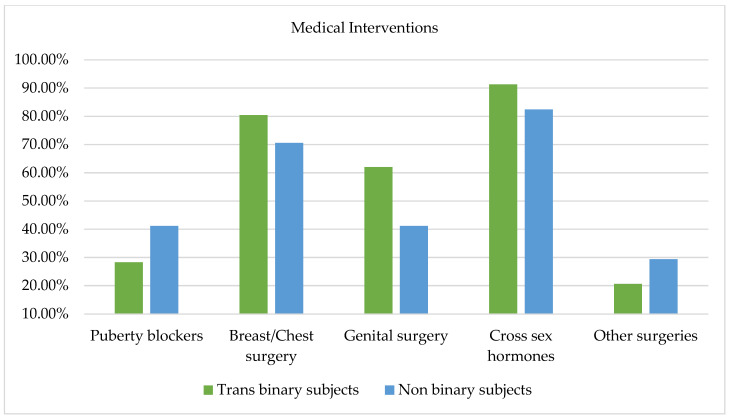
Desired medical interventions in trans binary and non-binary subjects.

**Figure 8:**
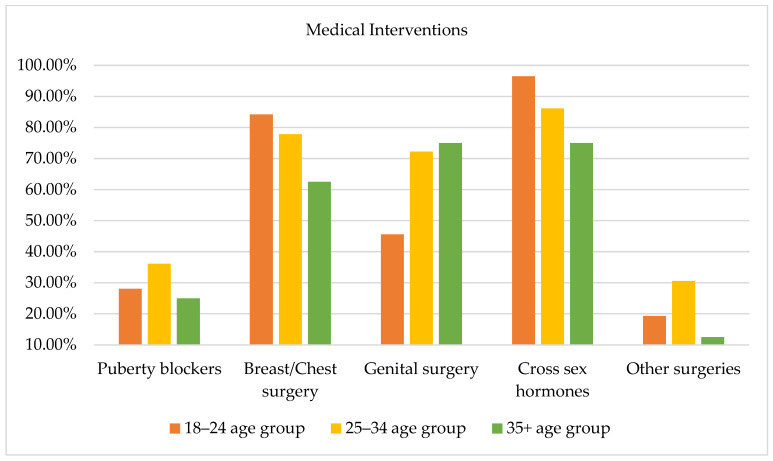
Desired medical interventions by age group.

**Table 1:** Demographic characteristics.

**Age Group**	
18–24 years	52.3% (*n* = 57)
25–34 years	33% (*n* = 36)
35+ years	14.7% (*n* = 16)
**Marital status**	
Single	57.8% (*n* = 63)
In a relationship	33.9% (*n* = 36)
Married	5.5% (*n* = 6)
Cohabitating	1.8% (*n* = 2)
Separated/divorced	0.9 (*n* = 1)
**Educational level**	
Secondary school	18.3% (*n* = 20)
High school	50.5% (*n* = 55)
Professional school	11% (*n* = 12)
University	18.3% (*n* = 20)
Other	0.9 (*n* = 1)
**School drop-out**	
Not a school drop-out	85.3% (*n* = 93)
School drop-out	11.9% (*n* = 13)
**Employment**	
Student	34.9% (*n* = 38)
Employed	31.2% (*n* = 34)
Unemployed	29.4% (*n* = 32)
Not specified	4.59% (*n* = 5)
**Household composition**	
Living with family	56.0% (*n =* 61)
Living with friends	1.8% (*n* = 2)
Living with partner/cohabitant	9.2% (*n* = 10)
Living alone	15.6% (*n* = 17)
Living with partner and children	3.7% (*n* = 4)
Other	11% (*n* = 12)
**Family events**	
Parental divorce	34.9% (*n* = 38)
No parental divorce	60.6% (*n* = 66)
**Children**	
Yes	2.8% (*n* = 3)
No	97.2% (*n* = 106)
**Hormone therapy**	
Undergoing hormone therapy	5.54% (*n* = 6)
Not undergoing hormone therapy	94.5% (*n* = 103)
**Psychotherapy**	
Psychotherapy prior to arrival	60.6% (*n* = 66)
Psychotherapy after arrival	50.5% (*n* = 55)

**Table 2:** Results of a chi-square test comparing trans binary and non-binary participants on gender fluidity.

	Fixed Gender Identity (No Time- or Context-Based Change)		Fluid Gender Identity (Context-Based Change)		Fluid Gender Identity (Time-Based Change)		Currently Exploring Gender Identity	
	Chi2	*p*	Chi2	*p*	Chi2	*p*	Chi2	*p*
Trans binary vs. non-binary	10.516	0.005	16.206	<0.001 *	28.568	<0.001 *	19.125	<0.001 *

* *p* < 0.001.

## References

[1] Mirabella M., Di Giannantonio B., Giovanardi G., Piras I., Fisher A.D., Lingiardi V., Chianura L., Ristori J., Speranza A.M., Fortunato A. (2023). Exploring Gender Diversity in Transgender and Non-Binary Adults Accessing a Specialized Service in Italy. Healthcare.

